# Escape of SARS-CoV-2 Variants KP.1.1, LB.1, and KP3.3 From Approved Monoclonal Antibodies

**DOI:** 10.20411/pai.v10i1.752

**Published:** 2024-09-30

**Authors:** Delphine Planas, Isabelle Staropoli, Cyril Planchais, Emilie Yab, Banujaa Jeyarajah, Yannis Rahou, Matthieu Prot, Florence Guivel-Benhassine, Frederic Lemoine, Vincent Enouf, Etienne Simon-Loriere, Hugo Mouquet, Marie-Anne Rameix-Welti, Olivier Schwartz

**Affiliations:** 1 Virus and Immunity Unit, Institut Pasteur, Université Paris Cité, CNRS UMR3569, Paris, France; 2 Vaccine Research Institute, Créteil, France; 3 Humoral Immunology Unit, Institut Pasteur, Université Paris Cité, Paris, France; 4 National Reference Center for Respiratory Viruses, Molecular Mechanisms of Multiplication of Pneumoviruses Unit, Institut Pasteur, Université Paris Cité, Paris, France; 5 G5 Evolutionary Genomics of RNA Viruses, Institut Pasteur, Université Paris Cité, Paris, France; 6 Bioinformatics and Biostatistics Hub, Paris, France; 7 Molecular Mechanisms of Multiplication of Pneumoviruses, Université Paris-Saclay, Université de Versailles St. Quentin, UMR 1173 (2I), INSERM; Assistance Publique des Hôpitaux de Paris, Paris, France

**Keywords:** SARS-CoV-2 variants, monoclonal antibodies, Sipavibart, Pemivibart, neutralization

## Abstract

**Background:**

First-generation anti-SARS-CoV-2 monoclonal antibodies (mAbs) used for prophylaxis or therapeutic purposes in immunocompromised patients have been withdrawn because of the emergence of resistant Omicron variants. In 2024, 2 novel mAbs, VYD222/Pemivibart and AZD3152/Sipavibart, were approved by health authorities, but their activity against contemporary JN.1 sublineages is poorly characterized.

**Methods:**

We isolated authentic JN.1.1, KP.1.1, LB.1, and KP.3.3 viruses and evaluated their sensitivity to neutralization by these mAbs in 2 target cell lines.

**Results:**

Compared to ancestral strains, VYD222/Pemivibart remained moderately active against JN.1 subvariants, with a strong increase of 50% Inhibitory Concentration (IC50), reaching up to 3 to 15 µg/mL for KP3.3. AZD3152/Sipavibart neutralized JN.1.1 but lost antiviral efficacy against KP.1.1, LB.1, and KP3.3.

**Conclusions:**

Our results highlight the need for a close clinical monitoring of VYD222/Pemivibart and raise concerns about the clinical efficacy of AZD3152/Sipavibart.

## INTRODUCTION

**Figure 1.**
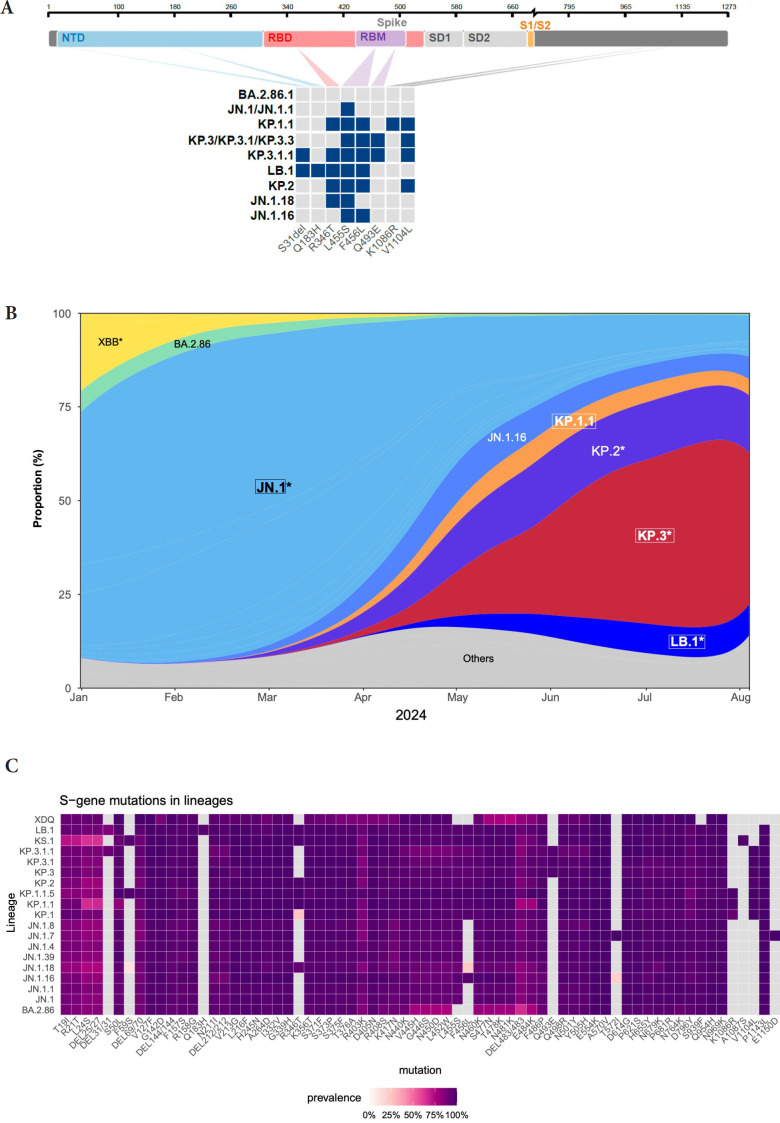
**Sequence and proportion of SARS-CoV-2 variants circulating in mid-2024.** (A) Spike mutations of SARS-CoV-2 variants relative to the spike domains of the BA.2.86.1 parental strain. JN.1, KP.1.1, LB.1, and KP.3.3 were further studied. The mutations in KP.2, JN.1.18, and JN.1.16 are also indicated. (B) Evolution of the prevalence of the main SARS-CoV-2 lineages from January to August 4, 2024, showing the expansion of JN.1-derived lineages. The star indicates the presence of different sublineages. The variants highlighted with a frame are analyzed in this study. Data from the GISAID EpiCoV database. (C) Comparison of spike mutations of selected SARS-CoV-2 variants using Wuhan_ Hu-1 (NC_045512) as reference. The color scale reflects the frequency of the mutations within lineages based on the data available on the GISAID EpiCOV database.

The JN.1 lineage arose in late 2023 and rapidly outcompeted previous SARS-CoV-2 variants [1]. Since then, JN.1 continued its evolution, with the appearance of sublineages carrying convergent mutations in the Spike (S) protein, notably F456L or R346T, and more recently S31del [2, 3] (Figure 1A,B). Sublineage specific mutations also appeared, such as Q493E. As of August 2024, the KP.1, KP.2, LB.1, and KP.3 variants, that carry various combinations of these substitutions, represented about 80% of sequenced circulating strains (Figure 1B,C). These mutations are collectively responsible for increased immune escape from previously infected and vaccinated populations [2, 3].

The sensitivity of KP.1, LB.1, and KP.3 variants to monoclonal antibodies (mAbs) developed for clinical use is poorly characterized. First-generation anti-S mAbs, previously approved by the Food and Drug Administration (FDA), the European Medicines Agency (EMA), or other agencies, had their authorization withdrawn after the emergence of Omicron variants, because of escape mutations in the receptor binding domain (RBD) of Spike protein. In 2024, novel mAbs, efficient against Omicron variants, have been tested in clinical trials or are available for clinical use in some countries [4]. These include AZD3152/Sipavibart, VYD222/Pemivibart, and SA55 that belong to different anti-RBD antibody classes and target distinct epitopes.

AZD3152 (or Sipavibart™) [5] was authorized in July 2024 by the EMA, for pre-exposure prophylaxis in patients with immunocompromising conditions and at high risk of developing severe COVID-19 [6]. However, AZD3152/Sipavibart-resistant viruses, carrying escape mutations at RBD positions 415, 456, and 458, have been described in pre-JN.1 lineages [5]. VYD222 (also termed Pemivibart or Pemgarda™) obtained an emergency use authorization by the FDA in March 2024, for pre-exposure prophylaxis in patients who are moderately to severely immuno-compromised [7]. VYD222/Pemivibart preserved *in vitro* efficacy against pre-JN.1 strains carrying the F456L mutation [7]. SA55 was isolated from a SARS-CoV-1 infected, SARS-CoV-2 vaccinated individual and displays a broad sarbecovirus neutralization profile, including JN.1, when used alone or in combination with another mAb (SA58) [8, 9]. SA55 has been tested in a clinical trial initiated in 2023 in China, in patients with hematological disorders who are persistently positive for SARS-CoV-2 [10].

Here, we isolated the main SARS-CoV-2 variants circulating in mid-2024 and tested their sensitivity to neutralization by a panel of mAbs.

## METHODS

### Virus Isolation

Viral strains were amplified through 1 or 2 passages on Vero E6 TMPRSS2 cells and 1 passage on IGROV-1 cells. Cells were plated in T75 flasks and cultivated in culture media (Dulbecco’s Modified Eagle Medium (DMEM), 10% fetal calf serum, and 1% Penicillin/Streptomycin) at 37°C, 5% CO2. Supernatants were harvested 2 or 3 days after viral exposure. Viral supernatants were sequenced directly from nasopharyngeal swabs and after isolation and amplification on IGROV-1 cells to confirm identity, the presence of specific mutations in the spike protein, and the absence of cell culture-derived mutations. The titration of viral stocks was performed on S-Fuse cells [1, 11, 12].

The D614G and JN.1 strains have been described [1, 13]. The KP.1.1 (hCoV-19/France/IDFRELAB-IPP05044/2024), LB.1 (hCoV-19/France/GES-RELAB-IPP04736/2024), and KP.3.3 strains (hCoV-19/France/BFC-IPP06087/2024) were isolated and amplified by the National Reference Center for Respiratory Viruses hosted by Institut Pasteur.

### Monoclonal Antibodies

S309 (or Sotrovimab) was previously described [14]. Codon-optimized synthetic DNA fragments coding for the immunoglobulin variable domains of SA55 (BD55-5514) [8], AZ3152/Sipavibart [5], and VYD222/Pemivibart were synthetized (GeneArt, Thermo Fisher Scientific) and cloned into human IgG1 expression vectors as previously described [15]. Recombinant IgG1 antibodies were produced by transient co-transfection of Freestyle™ 293-F suspension cells (Thermo Fisher Scientific) using the PEI-precipitation method and purified from culture supernatants by affinity chromatography using Protein G Sepharose^®^ 4 Fast Flow (GE Healthcare) as previously described [15].

### Cell Lines

IGROV-1 and S-Fuse (U20S) cells were previously described [1, 11]. Cells were regularly tested to confirm they were negative for mycoplasma.

### Virus Titration for Neutralizing Assay

Titration of viral stocks was performed on S-Fuse and IGROV-1 cells. Neutralization assays were conducted using a multiplicity of infection sufficient to produce about 200 syncytia/well with S-Fuse cells and achieve 40% of infected IGROV-1 cells.

### S-Fuse Neutralization Assay

U2OS-ACE2 GFP1-10 and GFP11 cells, also termed S-Fuse cells, become GFP+ when they are productively infected by SARS-CoV-2 [11, 13]. Cells were mixed (ratio 1:1) and plated overnight at 12 × 10^3^ per well in a μClear 96-well plate (Greiner Bio-One). The indicated SARS-CoV-2 strains were incubated with serially diluted monoclonal antibodies (mAbs) for 15 minutes at room temperature and added to S-Fuse cells. After 18 hours, cells were fixed with 2% PFA (Electron Microscopy Sciences, cat# 15714-S), washed, and stained with Hoechst (dilution of 1:1,000, Invitrogen, cat# H3570). Images were acquired using an Opera Phenix high-content confocal microscope (PerkinElmer). The number of GFP syncytia and the number of nuclei were quantified using Harmony software (PerkinElmer). The percentage of neutralization was calculated using the number of syncytia with the following formula: 100 × (1 – [value with mAb – value in “non-infected”]/[value in “no mAb” – value in “non-infected”]).

For each mAb, the half-maximal inhibitory concentration (IC50) in ng/mL was calculated with a reconstructed curve using the percentage of neutralization at each concentration.

### IGROV-1 Neutralization Assay

Sixteen hours before infection, 30 × 10^3^ cells per well were seeded in a μClear black 96-well plate (Greiner Bio-One). The indicated SARS-CoV-2 strains were incubated with serially diluted mAbs for 15 minutes at room temperature and added to IGROV-1 cells. After 24 hours, cells were fixed with 2% PFA (Electron Microscopy Sciences, cat# 15714-S). The cells were then intracellularly stained with anti-SARS-CoV-2 nucleoprotein (N) antibody NCP-1 (0.1 μg/mL) as described [1]. The staining was carried out in PBS with 0.05% saponin 1% BSA, and 0.05% sodium azide for 1 hour. Cells were then washed twice with PBS and stained with anti-IgG Alexa Fluor 488 (dilution 1:500, Invitrogen; cat# A11029) for 30 minutes before being washed twice with PBS. Hoechst 33342 (Invitrogen, cat# H3570) was added during the final PBS wash. Images were captured using an Opera Phenix high-content confocal microscope (PerkinElmer). The N-positive area and the number of nuclei were quantified using Harmony Software v4.9 (PerkinElmer). The percentage of neutralization was calculated using the N-positive area with the following formula: 100 × (1 – [value with mAb – value in “non-infected”]/[value in “no mAb” – value in “non-infected”]). For each mAb, the half-maximal inhibitory concentration (IC50) in ng/mL was calculated with a reconstructed curve using the percentage of neutralization at each concentration.

### Statistical Analysis

Figures were generated using Prism 9 (GraphPad Software). Statistical analysis was conducted using GraphPad Prism 9. Data are mean ±SD of 3 independent experiments.

### Lineage Monitoring

To visualize the evolution of the frequency of SARS-CoV-2 lineages, we analyzed the viral genomic surveillance data deposited in the GISAID database (https://www.gisaid.org; metadata downloaded on July 7, 2024) [16, 17]. The hierarchical relationships between lineages were retrieved from the pangolin GitHub repository (https://github.com/cov-lineages/pango-designation). We analyzed SARS-CoV-2 data collected from January 1, 2024, to August 4, 2024, using R 4.3 and ggplot 3.4.3. Mutations that are common and specific to lineages of interest were computed using the outbreak.info R package (https://outbreak-info.github.io/R-outbreak-info) [18].

### Data Availability

All data supporting the findings of this study are available within the article or from the corresponding author upon reasonable request without any restrictions. The sequencing data generated in this study have been deposited in the GISAID EpiCoV database.

## RESULTS

We examined the sensitivity of SARS-CoV-2 variants JN.1.1, KP.1.1, LB.1, and KP.3.3 to VYD222/Pemivibart, AZD3152/Sipavibart, and SA55. We included the ancestral D614G strain as control. We isolated KP.1.1, LB.1, and KP.3.3 (which carries the same S protein as KP.3) variants from nasal swabs of individuals with sequence-diagnosed infections. Sequences of outgrown viruses confirmed the identity of the variants (Figure 1A). The mAbs were not commercially available for research purposes. Therefore, we retrieved their sequences from public databases and produced biosimilar molecules. As an additional control, we used Sotrovimab™ that neutralizes several Omicron strains but not JN.1, and is no longer approved [1].

**Figure 2.**
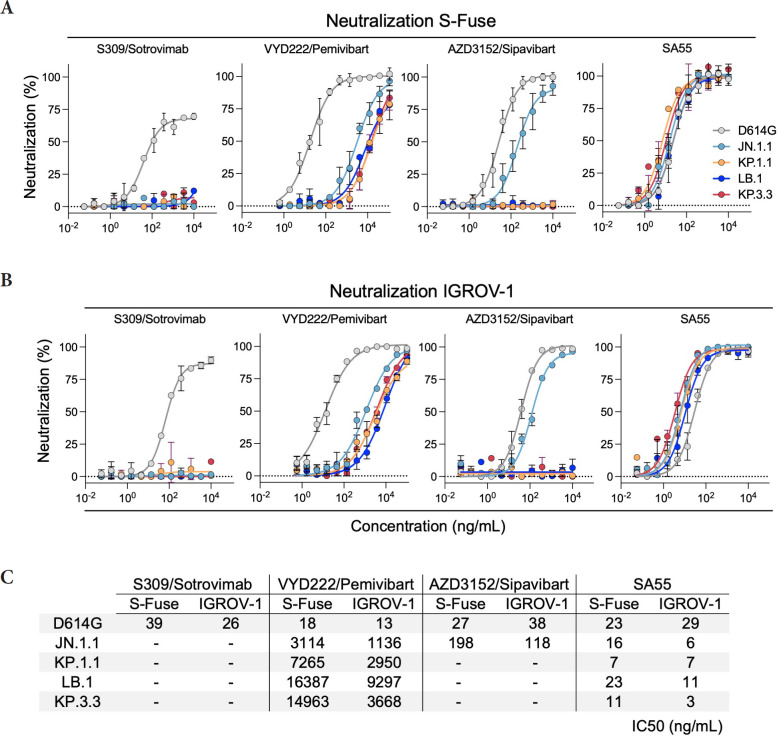
**Neutralization of SARS-CoV-2 variants circulating in mid-2024 by mAbs.** A. Neutralization curves of mAbs in S-Fuse cells. Dose-response analysis of neutralization of the indicated variants by S309/Sotrovimab, VYD222/Pemivibart, AZD3152/Sipavibart, and SA55. Data are presented as mean ± standard deviation of 2–3 independent experiments. B. Neutralization curves of mAbs in IGROV-1 cells. Dose-response analysis of neutralization of the indicated variants by S309/Sotrovimab, VYD222/Pemivibart, AZD3152/Sipavibart, and SA55. Data are presented as mean ± standard deviation of 2–3 independent experiments. C. EC50 values (in ng/mL) for each mAb against the indicated viral strains in the 2 cell lines. “- ”: no antiviral activity.

We measured the sensitivity of the viral isolates to mAbs first using S-Fuse cells as targets [12] (Figure 2A). These cells were engineered to express ACE2 and are thus sensitive to SARS-CoV-2 [1, 12]. The four mAbs efficiently neutralized D614G, with EC50s of 18–39 ng/mL, corresponding to those described in the literature. As expected, S309/Sotrovimab lost any activity against the 4 JN.1-derived strains. AZD3152/Sipavibart inhibited JN.1.1, with an EC50 of 198 ng/mL, but no longer neutralized KP.1.1, LB.1, and KP.3.3 (Figure 1B). The F456L substitution present in the 3 variants likely mediates this resistance. VYD222/Pemivibart was poorly active against JN.1.1 and displayed a decreased antiviral activity against KP.1.1, LB.1, and KP.3.3 (Fig. 2A). The EC50s reached up to 16,000 ng/mL, corresponding to up to 888-fold reduction of potency against the 4 variants compared to D614G. The antiviral activity of SA55 was preserved against the variants, with EC50s that remained remarkably low (7 to 23 ng/mL) (Figure 1B).

We did not isolate a KP.2 variant, but its profile of resistance is likely similar to KP.1, since their S only differ at position 1086, outside the RBD (Figure 1A).

We then sought to confirm these results using another cell line. We selected IGROV-1 cells, because they naturally express ACE2 and are highly sensitive to SARS-CoV-2, including Omicron and JN.1 variants [1]. The profile of neutralization of the 5 SARS-CoV-2 strains was similar in S-Fuse (Figure 2A) and IGROV-1 cells (Figure 2B). The IC50 were also in the same range in the 2 cell types (Figure 2C).

## DISCUSSION

Altogether, our results indicate that AZD3152/Sipavibart totally lost antiviral activity against the prevalent strains circulating in mid-2024, most likely because of the presence of the F456L substitution in Spike protein. VYD222/Pemivibart remains active against JN.1.1, KP.1.1, LB.1, and KP.3.3, with however a strong increase in IC50. The loss of activity of VYD222/Pemivibart has been recently reported in a preprint, using VSV-based pseudotypes [19]. As of August 2024, the KP3.1.1 variant that combines the F456L and Q493E mutations found in KP.3 and KP.3.3 with the S31 deletion found in LB.1 has been on the rise [19]. Future work will help to assess the sensitivity of the rapidly diversifying JN.1 family to these mAbs.

## CONCLUSION

Our *in vitro* results may not directly translate into clinical efficacy but raise concerns about the medical use of AZD3152/Sipavibart and warrant a close surveillance of Pemivibart, when most of the circulating strains totally or partially escape neutralization by the 2 antibodies. The mAb SA55 represents a promising alternative.
